# Volatile Flavor Compounds in Cheese as Affected by Ruminant Diet

**DOI:** 10.3390/molecules25030461

**Published:** 2020-01-22

**Authors:** Andrea Ianni, Francesca Bennato, Camillo Martino, Lisa Grotta, Giuseppe Martino

**Affiliations:** 1 Faculty of Bioscience and Technology for Food, Agriculture and Environment, University of Teramo, Via R. Balzarini 1, 64100 Teramo, Italy; andreaianni@hotmail.it (A.I.); francescabennato82@gmail.com (F.B.); lgrotta@unite.it (L.G.); 2 Istituto Zooprofilattico Sperimentale dell’Abruzzo e del Molise “G. Caporale”, Via Campo Boario, 64100 Teramo, Italy; c.martino@izs.it

**Keywords:** lactating ruminants, milk, cheese, volatile compound, lipolysis, proteolysis

## Abstract

Extensive research has been conducted concerning the determination and characterization of volatile compounds contributing to aroma and flavor in cheese. Considerable knowledge has been accumulated on the understanding of the mechanisms through which these compounds are formed during ripening, as well as on the optimization of the methodological approaches which lead to their detection. More recently, particular attention has been given to the aromatic properties of milk and cheeses obtained from lactating dairy ruminants fed experimental diets, characterized, for instance, by the addition of trace elements, natural supplements, or agricultural by-products rich in bioactive compounds. The purpose of this review is to summarize the major families of volatile compounds most commonly found in these types of dairy products at various ripening stages, describing in greater detail the role of animal diet in influencing the synthesis mechanisms most commonly responsible for cheese flavor determination. A large number of volatile compounds, including carboxylic acids, lactones, ketones, alcohols, and aldehydes, can be detected in cheese. The relative percentage of each compound depends on the biochemical processes that occur during ripening, and these are mainly mediated by endogenous enzymes and factors of bacterial origin whose function can be strongly influenced by the bioactive compounds taken by animals with the diet and released in milk through the mammary gland. Further evaluations on the interactions between volatile compounds and cheese matrix would be necessary in order to improve the knowledge on the synthesis mechanisms of such compounds; in addition to this, more should be done with respect to the determination of synergistic effects of flavor compounds, correlating such compounds to the aroma of dairy products.

## 1. Introduction

Chemical stability represents the fundamental characteristic of numerous processed foods. However, in the case of cheese, reference is made to a highly dynamic product from the biochemical point of view, especially in those cheeses subjected to ripening. During this period hundreds of volatile compounds (VOCs) can be produced, thus giving rise to flavors and odors that are characteristic of each cheese variety [1].

The main biochemical pathways that occur during the cheese ripening are represented by the metabolism of residual lactose, lactate, and citrate, lipolysis which is associated to the release of free fatty acids (FFAs), and proteolysis that is responsible for casein degradation to peptides with different molecular weights and free amino acids (FAAs). In addition, all the catabolic reactions against FFAs and peptides that give rise to a wide range of VOCs should be included [2].

In the last decades several studies have been conducted with the aim of investigating the specific mechanisms responsible for the production of sapid compounds in cheese during ripening. This approach was driven by the intention to obtain information on the flavor chemistry of many cheese varieties. An aspect to which less attention has been given regards the influence of the feeding strategies administered to ruminants on the volatile profile found in ripened dairy products. It is well known that by modifying animal diet, variations in the chemical-nutritional composition can be induced in milk. Consequently, the characteristics found in milk can be transferred in cheese, making available different substrates for the metabolic functions of starter or non-starter bacteria and for the activities of lipolytic or proteolytic enzymes of endogenous origin [3]. This means that volatile and sensory characteristics of ripened cheeses are largely defined by the technological approach and the initial chemical composition of the raw material [4]. The basic dietary factors that should be considered in ruminants for their effect on milk composition are represented by the fiber content, the ratio between forage and a concentrate (generally consisting of cereal and legume flours in addition to mineral and vitamin supplements), the carbohydrate composition of the concentrate, and the lipid amount, meal frequency, and intake [5]. Over time these aspects have been extensively characterized, especially with a view to obtaining a milk with a greater predisposition to be used for the production of manufactured products [6].

Numerous studies have focused their attention on the correlation between certain variations in the chemical composition of milk and the presence in the ruminant diet of specific classes of bioactive compounds, for instance polyphenols and terpenes, which can be mostly found in plants [7,8]. In this regard a mention should be made to the work of Walker et al. [9], who discussed the most relevant aspects able to induce effects on fatty acid composition of dairy cows’ milk. High intake of starch is associated with increased de novo synthesis of fat in the mammary gland, with a consequent increase in the milk of saturated fatty acids (SFAs). In contrast, dietary intake of higher concentrations of polyunsaturated fatty acids (PUFAs) was demonstrated to be effective in inducing higher concentrations of unsaturated fatty acids (UFAs), including conjugated linoleic acid (CLA). An increased intake of starch-based concentrates is instead responsible for the reduction in milk fat concentration, a phenomenon which can be attributed to variations in the balance between lipogenic and glucogenic volatile FAs of ruminal origin. However, reduced fat levels in milk are presumably dependent also on the increased production in the rumen of long-chain FAs containing a *trans*-10 double bond, specifically C18:1 *trans*-10 and C18:2 *trans*-10 *cis*-12, in response to feeding strategies characterized by increased concentrations of PUFAs and/or starch.

In this context, we should include all studies in which ruminant diets have been integrated with agro-industrial by-products and the effects on the chemical-nutritional composition of milk and cheeses have been evaluated. For instance, the supplementation of dairy ewes’ diet with an olive crude phenolic concentrate obtained from olive oil wastewater was demonstrated to be effective in inducing in milk an increase in concentration of polyunsaturated fatty acids [10]. A similar behavior was also observed by administering dairy cows with a diet enriched with dried grape pomace, the main by-product of the wine industry; in this study the authors also evidenced an improvement of the oxidative stability of ripened cheese [11]. In addition to this, grape pomace supplementation was also demonstrated to induce in cow’s milk a significant increase in concentration of lactose and β-lactoglobulin, although no effects were found for α-lactalbumin, albumin, and caseins [12]. Although the consideration may be speculative, it is conceivable that such a finding may derive from the ability of bioactive compounds of dietary origin to influence bovine gene expression. Indeed, a recent study has shown that 75 days of dietary supplementation with dried grape pomace were effective in inducing variations in the whole-transcriptome of Friesian calves. In that case the authors specifically focused their attention on the pathway of cholesterol biosynthesis, and correlated the observed molecular variations with the reduction in both serum cholesterol and lipid oxidation in carcasses [13].

More recently, a fair number of papers have been published concerning the influence of the feeding strategy on the volatile profile of ripened dairy products obtained from lactating ruminants. The objective of this review is therefore to reorganize, as much as possible, the findings in this research area, in order to obtain a clearer view on the possible correspondences between the type of administered diet and variations in concentration of specific VOCs found in dairy products during ripening. The discussion will be performed on the individual classes of compounds (carboxylic acids, aldehydes, lactones, ketones, alcohols, esters, and phenolic compounds), also giving a nod to the relevance of specific VOCs in flavor perception and summarizing, if appropriate, the principal biochemical pathways by which flavor compounds are produced and that could be influenced by the presence of specific bioactive compounds of dietary origin.

## 2. Biochemical Mechanisms Responsible for the Production of Volatile Flavor Compounds in Dairy Products

The biochemical mechanisms that characterize cheese ripening can be grouped into primary and secondary events. Primary events are represented by the metabolism of residual lactose, lactate, and citrate, as well as lipolysis and proteolysis. These events are then followed by secondary biochemical mechanisms involved in the metabolism of fatty acids and amino acids, which directly contribute to the synthesis of many VOCs, credited as having a high capacity to influence the cheese flavor [1,2,14].

### 2.1. Metabolism of Residual Lactose, Lactate, and Citrate

Lactose is the most represented carbohydrate in milk and is converted to lactate during the cheesemaking by the lactic acid bacteria (LAB), inducing a decrease in pH. In turn, lactate can be further processed by LAB in order to release formate, acetaldehyde, ethanol, and acetate [1,2]. With regard to citrate, the residue remaining in the curd can be converted by citrate-positive LAB into acetate and lactate after cheesemaking. This event is also responsible for the production of additional volatile compounds such as acetoin, 2,3-butanediol, diacetyl, and 2-butanone [15].

### 2.2. Metabolism of Free Amino Acids (FAA)

**Figure 1:**
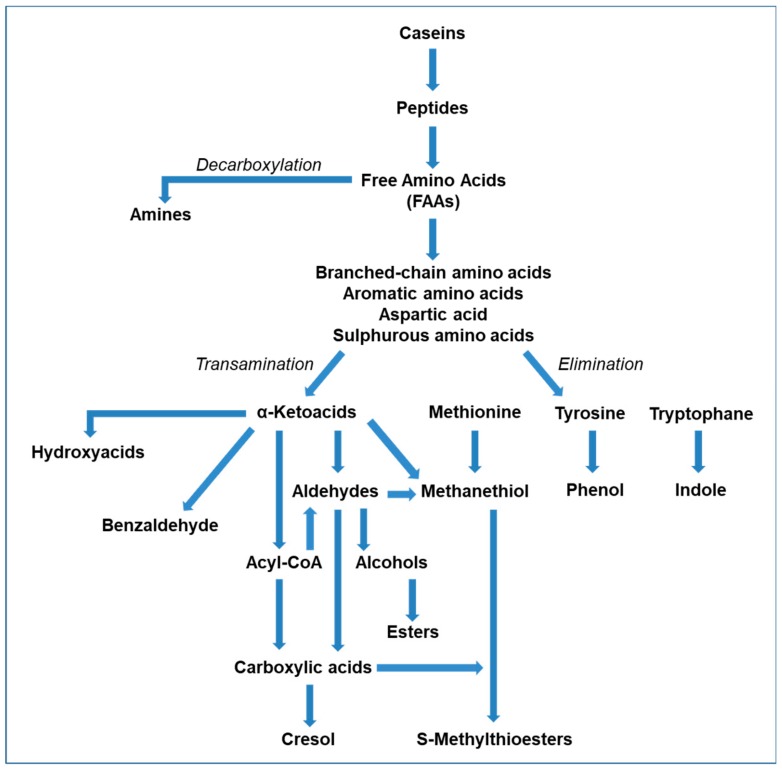
Schematic representation of the free amino acid (FAA) catabolism in cheese, modified from Bertuzzi et al. (2018).

The catabolism of FAAs represents the biochemical pathway mainly involved in the production of aldehydes, alcohols, carboxylic acids, amines, and sulfur compounds (Figure 1) [16,17].

The amino acid aminotransferase catalyzes a transamination reaction which leads to the conversion of aromatic amino acids, branched-chain amino acids, methionine, and aspartic acid into α-ketoacids. These compounds are then further metabolized to branched-chain and aromatic aldehydes, acyl-CoA, hydroxy acids, and methanethiol [16,18]. The production of 2-methylpropanal, 2-methylbutanal, and 3-methylbutanal is respectively due to the transamination of valine, isoleucine, and leucine, while the transamination reaction in which the substrate is represented by aspartic acid, is responsible for the release of oxaloacetate, which is in turn further converted into acetoin, diacetyl, or 2,3-butanediol [15,19]. Recently, the pivotal role of the aspartic acid transamination was demonstrated in the production of diacetyl in *Lactobacillus paracasei* [20]. Previously, in *Lactococcus lactis* var. *maltigenes* the existence of specific enzymatic pathways responsible for the production of phenylacetaldehyde and methional was observed, as a result of phenylalanine and methionine reduction, respectively [21].

With regard to aromatic aldehydes, these compounds are mainly formed starting from α-keto acids deriving from the benzaldehyde released by the spontaneous oxidation of tryptophan and phenylalanine. In this case it is therefore necessary to establish a condition causing a predisposition to a redox reaction, which is reported to be strongly influenced by the temperature, since an increase of this parameter involves catabolism acceleration [22]. Aldehydes represent the substrate of several dehydrogenases, which are able to convert such compounds to alcohols or to oxidize them into the corresponding carboxylic acids [16]. The metabolism of molds and yeast has been reported to be mainly involved in the biosynthesis of primary and aromatic alcohols, with the consequent release of corresponding carboxylic acids. In this regard, a study conducted by Yvon and Rijnen on *Geotrichum candidum* and yeasts isolated from Camembert allowed for the characterization of the mechanisms leading to the production of alcohols and carboxylic acids through FAA metabolism [23].

The excessive proteolysis in cheeses subjected to an uncontrolled ripening in terms of environmental conditions and duration leads to the formation of high concentrations of FAAs that can be decarboxylated, mainly by non-starter LAB, with the consequent release of biogenic amines, which are associated with poor flavor and potentially negative effects on consumer health. The most relevant biogenic amines are represented by histamine, tyramine, cadaverine, and putrescine, which are respectively synthesized starting from histidine, tyrosine, lysine, and ornithine [24].

In the context of FAA catabolism, a noteworthy aspect is also represented by the elimination reactions, which cleave the side chain of amino acids through a reaction catalyzed by a lyase. Over time substantial evidence has been collected about the fact that these reaction are associated to potential negative effects on flavor, as a consequence of the release of compounds such as p-cresol, phenethanol, and indole. This pathway also leads to the synthesis of methanethiol from methionine, which can be metabolized through a variety of different pathways. The major biosynthetic pathway in several strains is that of cystathionine, which involves the intervention of a cystathionine lyase. The further catabolism of methanethiol occurs through oxidative mechanisms performed by numerous LAB species, which are responsible for the production of dimethyldisulfide and dimethyltrisulfide. These compounds are reported to be characterized by low odor perception, thus markedly influencing the cheese flavor [25,26].

### 2.3. Metabolism of Free Fatty Acids (FFAs)

Lipolysis in dairy products is supported by the activity of lipases, microbial enzymes, enzymes of endogenous origin, and enzymes deriving from the added rennet pastes, which catalyze the triglyceride hydrolysis, with the consequent production of medium-chain (chain lengths up to 10 carbon atoms) and long-chain (chain lengths over than 10 carbon atoms) FFAs, di- and mono-glycerides, and glycerol [27].

The flavor properties of cheese are directly influenced by FFAs abundance and pH, and these parameters tend to influence each other. In presence of high pH values in cheese, the FFAs are reported to be less prone to the release of compounds capable of significantly influencing the flavor. Specifically, in this condition the FFAs are converted in non-volatile salts which induce the onset of unpleasant “soapy” aromatic notes. When pH is low, the FFAs are present in volatile form in the dairy matrix, and their excessive increase in concentration is generally effective in inducing a rancid taste [14].

**Figure 2:**
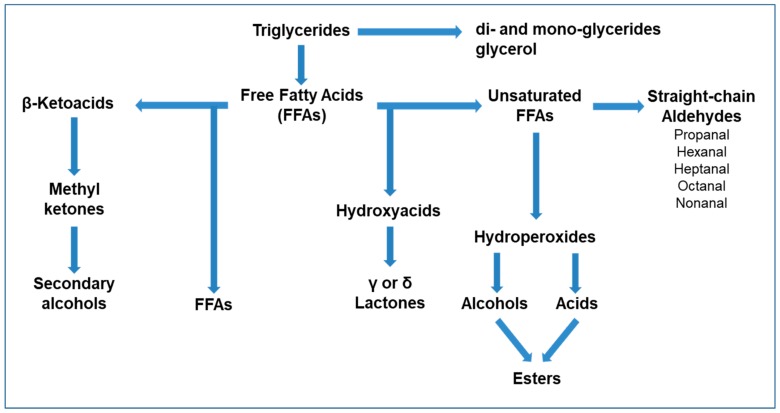
Schematic representation of free fatty acid (FFA) catabolism in cheese, modified from Bertuzzi et al. (2018).

As schematized in Figure 2, methyl ketones, secondary alcohols, straight-chain aldehydes, lactones, esters, and S-thioesters represent classes of VOCs partially deriving from the catabolism of FFAs, which therefore can contribute to the formation of cheese also indirectly as precursors of aromatic compounds [18,28]. FFAs can undergo oxidation, giving origin to β-ketoacids, which are converted to the corresponding methyl ketones through decarboxylation [17,27]. The biosynthetic pathway of methyl ketones is mainly associated to biochemical mechanisms performed by molds; however, hypotheses with regard to synthesis mechanisms induced by heating milk, or, alternatively, derived from a direct esterification of β-ketoacids [28] have been proposed. With regard to ketones, their possible overestimation in the volatile profile of dairy samples can occur as a consequence of the direct conversion of the β-ketoacids in the gas chromatograph inlet [29].

The enzymatic reduction of ketones leads to the production of secondary alcohols, a mechanism mainly attributed to molds (such as *Penicillium* spp.) which are specifically responsible for the production of 2-pentanol, 2-heptanol, and 2-nonanol in blue veined cheeses [27]. Such compounds are reported to minimally contribute to the cheese flavor, although the 2-heptanol was identified as a strong odorant in Gorgonzola and Grana Padano cheese [26].

Unsaturated free fatty acids and esterified fatty acids can undergo an auto-oxidation process through non-enzymatic mechanisms, releasing straight-chain aldehydes, mainly propanal, hexanal, heptanal, octanal, and nonanal, that are responsible for the so defined “green grass-like” aroma [27].

The synthesis of esters can occur by esterification, mediated by esterases which use alcohols and carboxylic acids as substrates, or through alcoholysis, which involves the activity of acyltransferases and leads to the synthesis of esters from alcohols, acylglycerols, or acyl-CoA mainly derived from the metabolism of FAAs and FFAs. The transfer to alcohols of fatty acyl groups from acylglycerols or acyl-CoA derivatives represents the main biosynthetic mechanism adopted by LAB to obtain esters. These compounds are associated to pleasant fruity notes able to reduce the sharpness and bitterness that occur in dairy products in which an increase in concentration of FFAs and amines is observed [26]. During esterification or alcoholysis the production of S-methyl-thioesters may occur, a phenomenon mainly correlated to the presence of methanethiol, therefore strictly dependent on the metabolism of specific bacterial species such as *Micrococcaceae Brevibacterium linens*, and *Geotrichum candidum*. S-methyl-thioesters can be alternatively released by the reaction between FFAs and methanethiol, and are commonly found on the surface of mold-ripened cheese and in blue veined cheeses, conferring strong odors with low threshold perception [30].

Lactones are produced by hydroxylated FFAs, which are integrated in milk triglycerides and released by reactions catalyzed by specific enzymes or induced by heating processes. In addition, hydroxylated FFAs can alternatively be produced by the catabolism of unsaturated fatty acids mediated by lipoxygenases and hydratases of microbial origin [28].

Unlike what has been reported for the other classes of compounds, phenols and terpenes can be identified in several varieties of dairy products, as a direct consequence of their presence in milk before the cheese-making. Phenolic compounds are mostly found in appreciable concentrations in goat and ewe milk, and while they are generally associated with pleasant aromatic notes, they tend to negatively affect the cheese flavor if present in excessive concentrations.

## 3. Major Volatile Flavor Compounds Found in Ripened Cheese and Influenced by Ruminant Diet

The lipolysis and the catabolism of fatty acids represent the most common biochemical mechanisms in cheese during ripening [27]. Therefore, the most represented family of VOCs in cheeses is usually that of carboxylic acids, generally composed of acids from C2 (acetic) to C10 or C12 (decanoic or dodecanoic) [31], followed by other classes of compounds such as aldehydes, lactones, ketones, alcohols, esters, and phenolic compounds. As summarized in Table 1, all these classes of compounds may undergo variations in quantity and composition, as a consequence of variations in the diet administered to ruminants.

### 3.1. Acids

Acetic acid can be synthesized by the catabolism of lactose, citrate, and FAAs, can alternately be derived from propionic fermentation, and is associated to pungent, vinegary, and acidic notes [1,32]. Ianni et al. [34] showed a significant decrease in concentration of this compound in fresh and 28-day ripened cheeses obtained from lactating cows fed for 60 days with 5% dietary supplementation of grape pomace, the major by-product of the oenological industry. A plausible explanation for this finding probably lies in the fact that grape pomace induced in milk an increase in concentration of long-chain fatty acids, making less likely the release of short-chain free fatty acids. In this regard, the study of Harper et al. [49] is of note, in which milk fat was substituted with various vegetable lipids in Romano and Cheddar cheeses. During the ripening process of cheese slurries, low molecular weight free fatty acids were formed, although the vegetable fats did not contain these compounds. As also discussed by Urbach [29], this interesting behavior was not fully characterized by authors from a microbiological and biochemical point of view, and it is therefore possible that the behavior observed may in part have been determined by exogenous factors.

Butanoic and hexanoic acids are considered to be the primary cause of strong and, in some cases, unpleasant odors defined as cheesy, rancid, and sweaty, and their tendency to increase in concentration during ripening in hard cheeses has been widely observed and characterized [50,51]. In a recent study conducted by Aprea et al. [33] a significantly lower concentration of both compounds was observed in ripened Montasio cheese obtained from Italian Simmental cows grazing in a pasture characterized by a nutrient-rich vegetation type. A similar behavior was also observed in other studies in which the ruminant diet was supplemented with plant matrices, which is particularly interesting from the biological point of view, due to the high content of bioactive compounds. Specifically, the reduction of butanoic and hexanoic acids in ripened cheeses was obtained by enriching the diet of Saanen goats with 1% of dried licorice root for 60 days [35], and by administering dietary grape pomace supplementation in lactating Friesian cows [34]. In contrary to the above reported, an increase in butanoic and hexanoic acid was evidenced in dairy products obtained from Friesian cows given dietary zinc supplementation. The particularity in this case lies in the fact that this finding was observed both in a 30-day ripened Caciotta cheese and in a fresh Italian dairy product, the Giuncata cheese, which was analyzed after 5 days of storage at 4 °C [36,37]. The general increase in concentration of FFAs, such as butanoic and hexanoic acids, is commonly explained by the extent of starter cell autolysis during cheese ripening, with the consequent release of enzymes, especially lipases, that promote lipolysis by cleaving the ester linkage between the fatty acid and the glycerol of the triacylglycerol [27]. The breaking of the bacterial envelope and the release of enzymatic factors into the extracellular environment is mediated by peptidoglycan hydrolases, commonly named autolysins, which are characterized by an N-terminal domain, a central catalytic domain, and a C-terminal domain containing a binding motif for zinc, which therefore represent a valuable cofactor [52]. The increase of FFAs in the presence of zinc may therefore depend by the ability of the trace element to favor bacterial autolysis in cheese. In the case of licorice root and grape pomace the opposite phenomenon was instead observed, presumably due to the ability of bioactive compounds deriving from these matrices to slow down the lipolytic action. In this regard it could be taken into account that lipase activity in cheese is strongly influenced by the concentration and type of fatty acids present in the reaction environment [3]. Indeed, the dietary intake of both licorice and grape pomace induced in milk significant variations in the fatty acid profile, with a presumable effect especially on lipases of endogenous origin. This interpretation could also be applied to the just-mentioned studies based on the use of zinc with respect to the reduced production of short-chain FFAs in dairy products, and an increase in concentration of long-chain fatty acids in milk and specifically vaccenic (C18:1 *trans*-11), oleic (C18:1 *cis*-9), linoleic (C18:2 *cis*-9, *cis*-12), and rumenic (C18:2 *cis*-9, *trans*-11) acids.

With regard to the longer chain carboxylic acids, the picture seems to appear more complex. In a study conducted on ripened goat cheese, evidence was found of a tendency for octanoic, decanoic and dodecanoic acids to increase in concentration after short aging periods (about 12 weeks), reaching concentrations well above the threshold values of aroma perception [53]. Octanoic acid is considered to be the main “goaty” compound in dairy products, and is reported to exhibit a waxy aroma that strongly contributes to the flavor of hard goat cheeses. Also, decanoic and dodecanoic acids undoubtedly influence the overall flavor of hard cheeses, and their increase is generally associated to soapy flavor [54]. With regard to the effect of the ruminant diet on the concentration of these compounds in dairy products during ripening, the study conducted by Bennato et al. [38] is noteworthy, as a reduction of dodecanoic acid was observed in a 60-day ripened cheese obtained from goats given dietary supplementation with extruded linseed. This plant matrix did not induce changes in the chemical composition of milk; the only variation was represented by the increase in concentration of linolenic acid (C18:3 *cis*9, *cis*12, *cis*15), which is known to be particularly represented in linseed. As previously reported, an effect of different acidic compositions of milk in influencing the activity of endogenous lipases during cheese ripening [3] could be hypothesized.

### 3.2. Aldehydes

Aldehydes are strongly flavored compounds and are commonly associated in foods to aroma defects referred to as oxidative rancidity [1,27]. These compounds are mainly released by the catabolism of FAAs and, in turn, represent the substrate for specific dehydrogenases responsible for the production of alcohols and carboxylic acids [17]. Aldehydes can also derive from non-enzymatic auto-oxidation reactions which lead to the degradation of unsaturated fatty acids, both free and esterified. These reactions do not occur with high frequency, since the cheese is characterized by a reducing environment. However, this event is responsible for the release of straight-chain aldehydes, which are reported to be associated with pleasant flavor notes [28]. The dairy matrices particularly rich in polyunsaturated fatty acids are therefore more prone to encountering oxidative phenomena able to produce aldehydes.

Recently, the enrichment of the ruminant diet with trace elements, such as zinc and selenium, resulted effective in inducing in milk, and consequently in cheese, an increase in concentration of PUFAs [39,40,41]. With regard to zinc, the authors specifically observed an increase in desaturation of stearic acid, and this finding was at least in part attributed to the role of zinc as a cofactor for a protease involved in the expression of stearoyl coenzyme A desaturase (SCD) in the mammary gland. SCD is reported to be an endoplasmic reticulum-bound enzyme responsible for the Δ^9^-desaturation of saturated fatty acyl-CoAs. The gene expression of this enzyme is mediated by the sterol response element binding proteins (SREBPs) which are activated by a metalloprotease (Site-2 protease) that needs zinc to perform its catalytic function [55,56]. In these studies, the analysis of volatile profile in dairy products did not evidence significant variations in the amount of total aldehydes. Authors discussed this finding by advancing the hypothesis of a role of zinc and selenium in curbing the oxidative damage, a conclusion also supported by the reduction of lipid oxidation evaluated by measuring in cheese the thiobarbituric acid reactive substances (TBARS). In this regard, zinc has been reported to inhibit lipid peroxidation in biological systems by competing with prooxidant metals (i.e., Cu and Fe) for binding sites, thus decreasing their ability to transfer electrons in a particular environment [57]. In the case of selenium, its antioxidant property lies in the ability to act as a scavenger of reactive oxygen-based radicals, with a direct effect in opposing the lipid oxidation in biological systems [58]. Although no differences were found in the total aldehyde content, interesting differences were observed at the level of individual compounds. The dietary zinc supplementation induced a significant increase in concentration of nonanal and hexanal in 120-day ripened Caciocavallo cheese and in 90-day ripened Pecorino cheese, respectively [39,40], whereas the selenium supplementation administered to Friesian cows was effective in inducing a decrease in hexanal and heptanal in samples of 30-day ripened Caciotta cheese [41]. Therefore, in light of what has been reported, dietary zinc enrichment seems to induce a better effect on the aromatic properties of ripened dairy products, since nonanal and hexanal, unlike other aldehydes, are commonly associated with pleasant herbal and slightly fruity notes [28].

### 3.3. Lactones

The main precursors of lactones are represented by hydroxylated FFAs which are incorporated in milk fat triglycerides and released as a result of enzymatic lipolytic mechanisms or the heating process. Hydroxylated FFAs can also be produced by the activities of lipoxygenases or hydratases of microbial origin, within the catabolism of unsaturated fatty acids. A reaction of one-step transesterification is effective in synthesizing lactones from hydroxylated FFAs [28]. These mechanisms heavily and quite positively affect the cheese flavor, since lactones are associated with very pronounced fruity notes, although they have been found to also contribute in cheese to the buttery character [59]. The synthesis of lactones leads to the release of α- and β-lactones that are reported to be highly reactive and unstable, while γ- and δ-lactones are stable and have been identified in several dairy products. In Cheddar cheese, the concentration of lactones rapidly increased in the early stages of the ripening, reaching levels well above their thresholds of flavor perception. δ-Octalactone was reported to be the most represented lactone in Parmigiano Reggiano cheese, while γ-decalactone, δ-decalactone, γ-dodecalactone, and δ-dodecalactone have been found in several French blue cheeses [60].

Also in this case, the studies previously cited have highlighted an active role of the ruminant diet in inducing a change in the relative concentration of this class of volatile compounds. In Caciocavallo cheese obtained from Friesian cows given zinc supplementation, there was evidence of different lactones: γ-nonalactone, γ-dodecalactone, δ-nonalactone, δ-decalactone, δ-dodecalactone, and δ-tetralactone. All these compounds went through a significant increase in concentration at the end of the 120 days of ripening [39]. No specific studies have been conducted on the effect of zinc in the biosynthetic pathway of lactones; however, as reported in the previous paragraphs, a role of the trace element in promoting the starter cells autolysis with consequent release of lipases in the dairy environment could be hypothesized [52]. This event has been reported to be responsible for the increase in concentration of FFAs, from which hydroxyacids, precursors of γ- and δ-lactones [28], are derived. The dietary zinc supplementation was also reported to induce an increase in concentration of lactones in samples of 30-day ripened Caciotta cheese, in which the compounds involved were however limited to δ-octalactone and δ-decalactone [36]. This phenomenon, involving δ-octalactone and δ-decalactone, has also been observed in samples of 30-day ripened Caciotta cheese obtained from lactating Friesian cows given dietary selenium supplementation [41]. This finding therefore suggests a common role of trace elements in favoring the biochemical mechanisms, especially of an enzymatic type, responsible for the synthesis of this class of VOCs.

Interestingly, lactones did not seem to undergo noteworthy variations in experimentations in which the diet of dairy goats and cows was supplemented with plant matrices such as linseed, licorice root, and grape by-products, rich in compounds credited of considerable interest from a biological point of view because of their well characterized anti-inflammatory and antioxidant properties [34,35,38]. An exception to this consideration is found in the study conducted by Castellani et al. [42], who administered to dairy cows a dietary supplementation of olive pomace, a by-product of the olive oil production, rich in fiber and unsaturated fatty acids. In samples of 30-day ripened Caciotta cheese, authors observed an increase in concentration of γ-dodecalactone and δ-octalactone, together with compounds belonging to other chemical classes such as 2-octenal and 1-hexanol.

### 3.4. Ketones and Alcohols

Ketones and alcohols mainly derive from biochemical mechanisms involving the lysis of triglycerides and the oxidation of saturated FFAs, with the consequent production of ketoacids that are decarboxylated to ketones which, in turn, can be reduced to obtain alcohols [61]. These compounds are mainly released by molds such as *Penicillium roqueforti* and *Penicillium camemberti*, which are responsible for typical odors that characterize the aroma of ripened blue veined cheeses [27]. In order to appreciate the potential contribution of these compounds to the cheese aroma, it could be useful to consider that in water, methyl ketones are reported to be characterized by perception thresholds that are quite low, ranging from 0.09 mg·100 g^−1^ for 2-heptanone and 4.09 to 50.0 mg·100 g^−1^ for 2-propanone [28].

In many studies on the volatile profile of cheeses, these classes of compounds are present in limited concentrations, precisely due to the fact that in the manufacturing of many dairy products molds are unwanted and strongly countered [29]. In addition to this, it should be mentioned that the concentration of ketones and alcohols does not seem to be particularly related to the degree of maturation of the cheeses, with a heterogeneous condition of complicated interpretation.

Stefanon and Procida [43] conducted a study aiming to evaluate the effects of including silage in dairy cow diet on the volatile profile of Montasio cheeses. During cheese ripening, significant variations were evidenced for ketones and mostly for the amount of total alcohols, with specific changes in concentration of ethanol, isobutanol, 1-penten-3-ol, and 2-methyl-1-butanol. Authors discussed these findings by assuming a direct effect of diet composition in affecting microbial and chemical fermentations in cheese during ripening rather than a transfer of selected compounds from milk. The Montasio cheese was also the subject of the research conducted by Bovolenta et al. [44], who performed evaluations on the volatile profile of cheese obtained by using raw milk coming from Italian Simmental cows grazing on two alpine pastures different for botanical composition. The “nutrient-poor pasture” resulted effective in inducing molding of the volatile profile of 60-day ripened cheese; in particular an overall increase in concentration of ketones, phenolic compounds, and terpenes was observed, with consequent slight effects noticed by panelists in the sensory analyses. With specific regard to terpenes, during cheese ripening differences were observed that the authors justified by taking into account the study of Belviso et al. [62] who demonstrated the ability of lactic acid bacteria isolated from cheese to influence the terpenoid biosynthesis.

An interesting behavior was recently observed in the volatile profile of Caciotta cheese obtained by enriching dairy cows diet with olive pomace. The dietary supplementation was effective in inducing a significant increase in FFAs, ester, and ketones in raw milk; however, following pasteurization and cheese-making, these differences disappeared both in the fresh and 30-day ripened dairy product [63]. Authors did not specifically investigate this phenomenon but hypothesized that the observed variations could at least in part derive from a change in the microbial population in pasteurized milk cheese, thus passing from a prevalence of lactic acid bacteria in raw milk to a greater concentration of the microbial genera used for pasteurized cheese manufacturing (*Lactococcus*, *Lactobacillus*, *Streptococcus*, and *Propionibacterium*).

Ianni et al. [45] compared the aromatic compounds of Caciocavallo cheeses obtained from Friesian cows fed a standard diet and a diet supplemented with selenium. Although the trace element did not induce differences in the chemical composition of milk and cheese, interesting variations were identified in the volatile profile of 120-day ripened cheeses, in which an increase in concentration of two methyl ketones (2-pentanone and 2-nonan-2-one) and a decrease of an alcohol (1-hexanol) were found. In this study changes in the family of ethyl esters were also highlighted, but no evaluations were executed on the hypothetical consumer acceptability of the experimental dairy product, since no sensory analyses were performed.

### 3.5. Esters

Esters represent a class of VOCs indirectly involved in the metabolism of FFAs [27]. Many of these compounds are reported to have low perception thresholds and are widely associated to a pleasant aroma characterized by sweet, fruity, and floral notes; furthermore, esters are appreciated for their role in stemming the bitterness and sharpness of cheeses, very often due to the high content of amines and FFAs [64].

Carpino et al. [46] analyzed the aroma-active compounds of Ragusano cheese obtained from dairy cows fed a total mixed ration (TMR) supplemented with native Sicilian pastures, in comparison with the same cheese obtained from cows fed only TMR. In samples of Ragusano cheese derived from native pasture 8 unique volatile flavor compounds were identified, among which 2 were esters, specifically geranyl acetate and [E]-methyl jasmonate. The latter compound represents a mediator of the physiological defense mechanisms adopted by plants subjected to stress induced by herbivorous insects. Specifically, when damage occurs plants produce VOCs reported to have detrimental effects on insect physiology [65]. The physical damage of the plant tissue entails the activation of the octadecanoid-lipoxygenase (LOX) pathway, responsible for the release of a wide range of lipid-derived VOCs. Therefore, it is conceivable that a small part of these compounds can be identified in raw milk and consequently in the cheese of ruminants fed with fresh pasture. The finding concerning the identification of unique odor-active esters was also found by analyzing the volatile profile of 60-day ripened goats’ milk cheese obtained from animals fed a dietary supplementation of extruded linseed, a well characterized plant matrix rich in linolenic acid (C18:3 *cis*-9, *trans*-12, *trans*-15). These esters, only detected in the “experimental” ripened cheese, were specifically the butanoic acid pentyl ester, the butyric acid 2-ethylhexyl ester, and the isopentyl hexanoate [54].

The previously mentioned addition of zinc to the diet of lactating dairy cows resulted effective in inducing noteworthy variations in volatile esters in derived dairy products. In 120-day ripened Caciocavallo cheese a marked increase in concentration of all the detected ethyl esters was shown, specifically ethyl butanoate, ethyl hexanoate, ethyl octanoate, ethyl nonanoate, ethyl decanoate, ethyl dodecanoate, ethyl tetradecanoate, and ethyl hexadecanoate. Interestingly, the last two compounds resulted only present in ripened cheese samples obtained from zinc feeding [39]. As previously reported, these data allow the discussion of a role of zinc in inducing an increase in lipolytic activity on the triglycerides present in the dairy matrix [52], with a consequent increase in concentration in the reaction environment of FFAs, contributing to the determination of the volatile profile not only directly, but also giving rise to other families of compounds, including esters [28]. In a 30-day ripened Caciotta cheese, the dietary zinc supplementation induced an increase in concentration of only two compounds, ethyl hexanoate and ethyl hexadecanoate, whereas no variations in this class of compounds were evidenced in Giuncata cheese, a fresh dairy product, that was analyzed after 5 days of storage at 4 °C [36,37]. In light of what has been just reported, it seems plausible that the observed increase in concentration of volatile ethyl esters is related to the length of the maturing period, although this consideration should be properly verified. As a partial support to the discussion, in one study lactating ewes were administered a zinc-enriched diet. In addition, samples of Pecorino cheese matured for 90 days showed an increase in concentration of two ethyl esters, specifically ethyl butanoate and ethyl hexanoate. In this case a slight but still significant reduction in concentration of ethyl octanoate was also reported [40].

### 3.6. Phenolic Compounds

Phenolic compounds are secondary metabolites of plants to which interesting properties are attributed from the biological point of view [66]. For that reason, over time great interest has been given to the development of functional dairy products containing specific phenolic compounds, such as catechin, tannic acid, hesperetin, and flavones, or natural crude compounds, for instance grape extract, green tea extract, and dehydrated cranberry powder [67].

Their presence in animal products can also derive from the direct transfer of these compounds from green herbage, or the synthesis by rumen bacteria which are reported to be mainly responsible for the lignin breakdown into monomeric phenols, through a mechanism characterized by decomposition of benzyl ether bonds of lignin polymers under anaerobic conditions [68]. Previous studies focusing on the evaluation of meat quality evidenced the presence of specific phenolic compounds in ruminant fat as a consequence of the ingestion of higher percentages in green herbage than in grain-based diets; specifically, the identification of 4-methylphenol in ruminant fat was reported to be positively affected by grazing [69,70].

As reported by O’Connell and Fox [71], the majority of phenolic volatile compounds identified in milk and dairy products are strictly related to the diet administered to ruminants, although a proportion may represent the product of FFA catabolism, preferably exploiting tyrosine as a precursor. In another study, in which lactating Friesian cows received a diet enriched with olive pomace, in pasteurized milk cheeses an increase in phenolic compounds was observed, specifically phenylacetaldehyde and 2-phenylethyl alcohol, both derived from the catabolism of phenylalanine. Authors discussed the finding by assuming a non-enzymatic Strecker degradation of phenylalanine or by enzymatic transamination of phenylalanine as an imide that is subsequently degraded to give phenylacetaldehyde, that, in turn, undergoes reduction to produce 2-phenylethyl alcohol [72].

With specific regard to cow milk, a study conducted by Villeneuve et al. [47] showed higher concentrations of toluene in samples obtained from cows on pasture, in comparison with milk samples collected from animals fed hay and silage. Authors discussed this finding by advancing the hypothesis of a greater degradation of β-carotene in forages such as silage or hay subjected to wilting and sun curing following harvesting [73]. Therefore, authors concluded that cows on pasture presumably consumed more β-carotene, explaining the significant increase in milk of toluene concentration. To better understand this finding, the study conducted by Contarini et al. [74] should be taken into account, where the effect of different heat treatments on the volatile profile of milk by applying a dynamic headspace capillary gas chromatography coupled with multivariate statistical approach was studied. Also in this work, it was assumed that the identification of toluene in raw milk was the consequence of β-carotene degradation. Furthermore, it was evidenced that the identification of toluene in milk, together with 2-pentanone, 2-heptanone, pentanal, and 3-methylbutanal, was effective in discriminating in-bottle sterilized milk (in which these compounds are more greatly represented) from pasteurized samples. 

Other studies confirmed that by feeding cattle with high levels of particular crops, other phenolic compounds may also be detected in ruminant milks, such as ptaquiloside, a norsesquiterpene from bracken (*Pteridium aquilinum*), or genistein and daidzein (derived from clover) [48].

## 4. Conclusions

In this review, the main biochemical mechanisms characterizing dairy products during ripening have been recalled, and the influence of different feeding strategies on the production and relative concentrations of various VOCs in fresh and ripened cheeses has been discussed.

Despite the large amount of research activity, to date the influence of certain dietary strategies on the quality of dairy products has not been well characterized, and there is a lack of findings useful to establish VOCs directly transferred from feeds to animal products that could be used for authenticity studies in order to discriminate milk samples or fresh and ripened dairy products. Furthermore, it should be also kept in mind that there is considerable variability induced by the cheese manufacturing process (heating, starter cultures type, ripening conditions), which could eliminate some of the VOCs present in milk. This remains an interesting challenge for researchers in the field of animal production.

## Figures and Tables

**Table 1:** Summary of the most relevant variations found in different dairy products obtained from ruminants fed experimental diets.

VOC Family	Dietary Supplement (Ruminant)	Type of Dairy Product	Effects	Ref.
Carboxylic acids	Dried grape pomace (Friesian cows)	Fresh and 28-day ripened Caciotta cheese	↓ Acetic acid	[32]
	Nutrient-rich pasture (Simmental cows)	12-month ripened Montasio cheese	↓ Butanoic acid ↓ Hexanoic acid	[33]
	Dried grape pomace (Friesian cows)	28-day ripened Caciotta cheese		[34]
	Dried licorice root (Saanen goats)	Fresh and 30-day ripened Caciotta cheese	↓ Hexanoic acid	[35]
	Organic zinc (Friesian cows)	30-day ripened Caciotta cheese	↑ Butanoic acid ↑ Hexanoic acid	[36]
		5-day stored Giuncata cheese		[37]
	Extruded linseed (Saanen goats)	60-day ripened Caciotta cheese	↓ Dodecanoic acid	[38]
Aldehydes	Organic zinc (Friesian cows)	120-day ripened Caciocavallo cheese	↑ Nonanal	[39]
	Organic zinc (half-breed ewes)	90-day ripened Pecorino cheese	↑ Hexanal	[40]
	Organic selenium (Friesian cows)	30-day ripened Caciotta cheese	↓ Hexanal ↓ Heptanal	[41]
Lactones	Organic zinc (Friesian cows)	120-day ripened Caciocavallo cheese	↑ γ-nonalactone ↑ γ-dodecalactone ↑ δ-nonalactone ↑ δ-decalactone ↑ δ-dodecalactone ↑ δ-tetralactone	[39]
		30-day ripened Caciotta cheese	↑ δ-octalactone ↑ δ-decalactone	[36]
	Organic selenium (Friesian cows)	30-day ripened Caciotta cheese		[41]
	Dried olive pomace (Friesian cows)	30-day ripened Caciotta cheese	↑ γ-dodecalactone ↑ δ-octalactone	[42]
Ketones and Alcohols	Silages (Simmental cows)	68-day, 200-day and 360-day ripened Montasio cheese	↑ acetone ↑ 2-3-butanedione ↑ 2-butanone ↑ 2-hexanone ↑ 2-heptanone ↑ 2-methyl-1-butanol	[43]
	Nutrient-rich vs nutrient-poor pasture (Simmental cows)	60-day ripened Montasio cheese	↑ 2-Propanone ^1^ ↑ 2-Hepta-none ^1^ ↑ 2-Undecanone ^1^ ↑ 3-Methyl-1-butanol ^2^	[44]
	Organic selenium (Friesian cows)	120-day ripened Caciocavallo cheese	↑ 2-pentanone ↑ 2-nonan-2-one ↓ Hexanol	[45]
Esters	TMR + native pasture (dairy cows)	Ragusano Cheese	Geranyl acetate ^3^ [E]-Methyl-jasmonate^3^	[46]
	Organic zinc (Friesian cows)	120-day ripened Caciocavallo cheese	↑ Ethyl butanoate ↑ Ethyl hexanoate ↑ Ethyl octanoate ↑ Ethyl nonanoate ↑ Ethyl decanoate ↑ Ethyl dodecanoate ↑ Ethyl tetradecanoate ↑ Ethyl hexadecanoate	[39]
		30-day ripened Caciotta cheese	↑ Ethyl hexanoate ↑ Ethyl hexadecanoate	[36]
Phenolic compounds	Pasture (dairy cows)	Raw milk	↑ Toluene	[47]
	Crops (dairy cows)		↑ Ptaquiloside ↑ Genistein ↑ Daidzein	[48]

VOC = volatile compound; TMR = total mixed ration; ↑ = Increase in concentration; ↓ = Decrease in concentration. ^1^ Data referred to cheese obtained from cows fed the nutrient-poor pasture. ^2^ Data referred to cheese obtained from cows fed the nutrient-rich pasture. ^3^ Compounds only found in cheeses obtained from cows fed the experimental feeding strategy.
